# Development of a nested PCR assay for specific detection of *Metschnikowia bicuspidata* infecting *Eriocheir sinensis*

**DOI:** 10.3389/fcimb.2022.930585

**Published:** 2022-07-22

**Authors:** Jie Bao, Ye Chen, Yuenan Xing, Chengcheng Feng, Qingbiao Hu, Xiaodong Li, Hongbo Jiang

**Affiliations:** ^1^ Aquaculture Department, College of Animal Science and Veterinary Medicine, Shenyang Agricultural University, Shenyang, China; ^2^ Key Laboratory of Livestock Infectious Diseases in Northeast China, Ministry of Education, Shenyang Agricultural University, Shenyang, China

**Keywords:** *Metschnikowia bicuspidata*, *Eriocheir sinensis*, hyphally regulated cell wall protein, nested PCR, milky disease

## Abstract

In recent years, the “milky disease” caused by *Metschnikowia bicuspidata* has seriously affected the *Eriocheir sinensis* culture industry. Discovering and blocking the transmission route has become the key to controlling this disease. The existing polymerase chain reaction (PCR) detection technology for *M. bicuspidata* uses the ribosomal DNA (rDNA) sequence, but low sensitivity and specificity lead to frequent false detections. We developed a highly specific and sensitive nested PCR method to detect *M. bicuspidata*, by targeting the hyphally regulated cell wall protein (HYR) gene. This nested HYR-PCR produced a single clear band, showed no cross-reaction with other pathogens, and was superior to rDNA-PCR in specificity and sensitivity. The sensitivity of nested HYR-PCR (6.10 × 10^1^ copies/μL) was greater than those of the large subunit ribosomal RNA gene (LSU rRNA; 6.03 × 10^4^ copies/μL) and internal transcribed spacer (ITS; 6.74 × 10^5^ copies/μL) PCRs. The nested HYR-PCR also showed a higher positivity rate (71.1%) than those obtained with LSU rRNA (16.7%) and ITS rDNA (24.4%). In conclusion, we developed a new nested HYR-PCR method for the specific and sensitive detection of *M. bicuspidata* infection. This will help to elucidate the transmission route of *M. bicuspidata* and to design effective management and control measures for *M. bicuspidata* disease.

## 1 Introduction

*Metschnikowia bicuspidata* (Ascomycota, Saccharomycetales) is a pathogenic yeast fungus that infects several economically important aquatic organisms, including shrimps: *Macrobrachium rosenbergii*, *Palaemonetes sinensis*, and *Exopalaemon carinicauda*; crabs: *Portunus trituberculatus* and *Eriocheir sinensis*; fish: *Oncorhyncus tshawytscha*; and bait organisms such as *Daphnia* and *Artemia* (Codreanu and Codreanu-Balcescu, 1981; Lu et al., 1998; Moore and Strom, 2003; Wang et al., 2008; Jiang et al., 2021; Cao et al., 2022; Ma et al., 2022; Zhao et al., 2022). *M. bicuspidata* can cause high mortality in crustaceans. Chen et al. (2007) found that the cumulative mortality of *M. rosenbergii* infected with *M. bicuspidata* could be as high as 95%. Xu (2005) found that the mortality rate of infected *P. trituberculatus* in Zhoushan City could reach 100%. In recent years, numerous *E. sinensis* farms in northern China have experienced severe *M. bicuspidata* infection, and adult crabs from overwintering ponds have shown infection rates exceeding 30% (Sun et al., 2022). The typical symptom of diseased *E. sinensis* is milky white and non-coagulant hemolymph, which crab farmers have aptly named “milky disease”. Infected crabs exhibit weakened vitality, lose appendages easily, and eventually die from organ failure (Bao et al., 2021; Zhang et al., 2021). As *E. sinensis* is widely traded in the national market, *M. bicuspidata* has infected cultured *E. sinensis* in numerous provinces and cities (Xu et al., 2021; Sun et al., 2022), which has caused great harm to the *E. sinensis* culture industry.

*M. bicuspidata* is a fungal organism with a thick spore wall and a strong resistance to the environment and drugs. Currently, there is no effective drug to treat *M. bicuspidata* infection. Therefore, strengthening prevention has become the key to controlling this disease. Although *M. bicuspidata* cannot spread vertically, it can rapidly spread through water, cannibalization, and co-habitation (Jiang et al., 2022) as well as through the food chain (Moore and Strom, 2003), which makes prevention difficult. Therefore, it is necessary to establish an accurate and sensitive detection technology to ensure that environmental organisms, water, sediment, stocking seedlings, and feed do not carry pathogens. The traditional pathogen detection method mainly uses the pathological symptoms of the host and microscopic observation of pathogen morphology. However, typical pathological symptoms only appear when the infection is severe. In the initial stage of infection, there are no apparent symptoms and detection under a microscope is difficult. Even if a small population of yeast can be observed, microscopy techniques cannot confirm whether it is *M. bicuspidata*. Molecular biology detection methods, such as polymerase chain reaction (PCR), have the advantages of simple operation, strong specificity, and the ability to identify species by sequencing (Nguyen et al., 2014; Fernández-Álvarez et al., 2019; Torres‐Corral and Santos, 2021). They are often used as detection methods for *M. bicuspidata* for diagnosis and epidemiological investigation (Ma et al., 2022; Sun et al., 2022).

Currently, all molecular tools for the detection of *M. bicuspidata* target the ribosomal DNA (rDNA) sequence, and primers are mainly designed using the large subunit ribosomal RNA gene (LSU rRNA) and internal transcribed spacer (ITS) rDNA sequences (Bao et al., 2021; Zhang et al., 2021). These primers are universal for yeasts and play an important role in pathogen identification (Mannarelli and Kurtzman, 1998; Fujita et al., 2001). To our knowledge, no other *M. bicuspidata* detection methods have been reported to date. However, primers for the rDNA sequence are likely to cross-amplify with other similar microorganisms, resulting in false-positive results because of low specificity (**Table 1**). The amplified product must be sequenced and compared to confirm whether it is from *M. bicuspidata*. In addition, conventional PCR has low detection sensitivity and cannot accurately detect light infections in the early stage of disease. Therefore, we have developed a new method to detect *M. bicuspidata* using nested PCR of the hyphally regulated cell wall protein gene (HYR). Based on the conventional PCR method, nested PCR uses two pairs of specific primers. The specificity and sensitivity of detection is improved through two rounds of amplification, which overcomes the problem of nonspecific amplification with the first pair of primers. Nested PCR is a low-cost, highly specific, and highly sensitive detection method that is often used for pathogen identification and detection (Manjanaik et al., 2005; Han et al., 2018; Cowley et al., 2019). The establishment of nested HYR-PCR technology provides a useful tool to accurately diagnose whether *E. sinensis* is infected with *M. bicuspidata*, especially in the early stages of infection, which will improve the prevention and treatment of this disease.

**Table 1:** Multiple sequence alignment analysis of target gene sequence for detection of *Metschnikowia bicuspidata*.

Gene sequence	Yeast species			% Identity		Accession No.
ITS	*Metschnikowia bicuspidata*			–		MT856369.1
	*Metschnikowia australis*			94.46		MH447359.1
	*Saccharomycetales* sp.			99.78		AB726734.1
	*Metschnikowia kamienskii*			98.90		KY108479.1
	*Metschnikowia* sp.			94.00		JQ857002.1
	*Metschnikowia zobellii*			92.19		U44823.1
	*Metschnikowia reukaufii*			90.28		MH047200.1
	*Metschnikowia* sp.			90.18		OM802671.1
	*Metschnikowia* sp.			94.15		KX773542.1
	*Metschnikowia* sp.			93.39		KC580664.1
	*Metschnikowia* sp.			93.55		KX773564.1
LSU rRNA	*Metschnikowia bicuspidata*			–		MT845876.1	
	*Metschnikowia australis*			91.39		MK085106.1	
	*Metschnikowia* sp.			90.20		MZ798278.1	
	*Metschnikowia cibodasensis*			90.18		AB236924.1	
	*Candida gelsemii*			90.13		DQ988045.1	
	*Metschnikowia reukaufii*			89.92		MH047200.1	
	*Metschnikowia gelsemii*			89.14		NR_164510.1	
	*Metschnikowia chrysomelidarum*			88.86		KY102031.1	
	*Metschnikowia maroccana*			88.52		MG993180.1	
	*Metschnikowia vanudenii*			88.71		MN128580.1	
	*Metschnikowia koreensis*			94.02		MN861602.1	

ITS, internal transcribed spacer; LSU rRNA, large subunit ribosomal RNA.

## 2 Materials and methods

### 2.1 Tested strains

*M. bicuspidata* was represented by the strain (LNES0119) preserved in our laboratory. The cells were streak cultured in Bengal red agar for activation. After culturing upside down in an incubator at 28°C for 48 h, a single colony was streak cultured again in Bengal red agar for 48 h at 28°C. Subsequently, the cultured colonies were extracted using a DNA Extraction Kit (Tiangen Biotech Co., Ltd., Beijing, China) and the products were stored in a refrigerator at –20°C. DNA samples of *Staphylococcus aureus*, *Enterocytozoon hepatopenaei*, *Hepatospora eriocheir*, *Microsporidia* sp., white spot syndrome virus, and *Vishniacozyma victoriae* (syn. *Cryptococcus victoriae*) were preserved in our laboratory for primer specificity detection.

### 2.2 Establishment of nested HYR-PCR detection system for *metschnikowia bicuspidata*

#### 2.2.1 Primer design

According to the genome reference sequence of *M. bicuspidata* published in NCBI, the specific hyphally regulated cell wall protein gene (HYR, Sequence ID: XM_018855835.1) was selected as the template. A pair of specific primers, P1/P2, were designed as the external primers for the first round of nested HYR-PCR amplification, and primers PN1/PN2 were designed as the internal primers for the second round of amplification (**Table 2**). The reaction procedure for the two rounds of nested HYR-PCR is shown in **Table 3**. Primers NL1/NL4 based on LSU rRNA (O'Donnell, 1993) and ITS1/ITS4 (White et al., 1990) based on ITS rDNA are used as the conventional PCR primers for comparison (**Table 2**).

**Table 2:** Primer sequences and amplified product fragments.

Primer type	Primer name	Primer sequence 5′-3′	Fragment size (bp)
First round of nested HYR-PCR	P1/P2	P1: AGCCTGGTCTTTGTAATG	493
		P2: ACTCCCTTGTTGGTGATA	
Second round of nested HYR-PCR	PN1/PN2	PN1: TTAGAGGGACTTCTCATTTGT	226
		PN2: CTTTAGCGTCAATATCGTAGA	
LSU rRNA	NL1/NL4	NL1: GCATATCAATAAGCGGAGGAAAAG	574
		NL4: GGTCCGTGTTTCAAGACGG	
ITS	ITS1/ITS4	ITS1: TCCGTAGGTGAACCTGCGG	394
		ITS4: TCCTCCGCTTATTGATATGC	

HYR, hyphally regulated cell wall protein; PCR, polymerase chain reaction.

**Table 3:** Nested PCR system.

Composition	First round reaction system	Second round reaction system
2 × *Taq* Master Mix	12 μL	12 μL
Upstream primer	0.5 μL	0.5 μL
Downstream primer	0.5 μL	0.5 μL
DNA template	2 μL	–
ddH_2_O	10 μL	10 μL
First round PCR products	–	2 μL
Total system volume	25 μL	25 μL

#### 2.2.2 Optimization annealing temperature of nested HYR-PCR

First, four temperature gradients (45, 50, 55, and 60°C) were set to optimize the annealing temperature of the external primers (P1/P2) and internal primers (PN1/PN2). The PCR conditions were as follows: pre-denaturation at 95°C for 10 min; 35 cycles of denaturation at 95°C for 1 min, annealing temperature for 45 s, and extension at 72°C for 1 min; and final extension at 72°C for 10 min. A 5 µL aliquot of the PCR product was electrophoresed for 30 min (120 V) in a 1.5% agarose gel, and the results were observed and photographed using a gel imaging system (BG-gdsAUTO520, Baygene Biotech Company Limited, Beijing, China).

#### 2.2.3 Nested HYR-PCR specificity identification

*M. bicuspidata* was set as a positive control and ddH_2_O as a negative control, and DNA samples from *S. aureus*, *E. hepatopenaei*, *H. eriocheir*, *Microsporidia* sp., white spot syndrome virus, and *V. victoriae* were used as specific identification templates. The external primers with optimized annealing temperature were used for the first round of PCR amplification. The first round PCR products were diluted 1000 times with ddH_2_O, and 2 μL of this dilution was used as the DNA template for the second round of PCR. The PCR procedure was as described in section 2.2.2.

#### 2.2.4 Nested HYR-PCR sensitivity identification

The template DNA of *M. bicuspidata* was used for PCR amplification with the primers P1/P2, NL1/NL4, and ITS1/ITS4. PCR products were recovered using a FastPure Gel DNA Extraction Mini Kit (Tiangen Biotech Co., Ltd., Beijing, China). The DNA fragments were then ligated to the pMD-19-T vector (Takara, Shiga, Japan). The recombinant plasmid was transferred into *E. coli* DH5α cells, and positive colonies were identified by PCR (Sangon, Shanghai, China). The recombinant plasmid was extracted using the FastPure Plasmid Mini Kit (Vazyme, Nanjing, China) and the concentration was measured using an ultramicro spectrophotometer (K5500, Beijing Kaiao Technology Development Co., Ltd., Beijing, China). The plasmid copy number was calculated and used as the plasmid standard. The concentration and copy number of the plasmids for each pair of primers are shown in **Table 4**. The plasmid standard was diluted step by step 10 times, and a total of 8 concentration gradients were set as amplification templates (**Table 4**). DNA from *M. bicuspidata* was used as the positive control, and ddH_2_O was used as the negative control. The reaction procedure was as described in section 2.2.2.

**Table 4:** Plasmid standard concentration and copy number of amplification products with different primers.

Primers	P1/P2	NL1/NL4	ITS1/ITS4
Concentration (ng/μL)	21.3	21.6	22.8
Copy number (copies/μL)	6.10 × 10^9^	6.03 × 10^9^	6.74 × 10^9^
Concentration gradient	6.10 × 10^8^∼6.10 × 10^1^	6.03×10^8^∼6.03 × 10^1^	6.74 × 10^8^∼6.74 × 10^1^

PCR products of eight concentration gradients in the first round were diluted 1000 times, and then 2 μL of this dilution was used as the template for the second round of PCR. The reaction system and reaction conditions remained unchanged.

### 2.3 Clinical samples testing

The crabs used for infection detection were purchased from an infested farm in Panjin City. Ninety crabs were randomly selected and dissected to obtain hepatopancreas tissue after ice anesthesia for 5 min. DNA was extracted using the Marine Animal Tissue DNA Extraction Kit (Tiangen Biotech Co., Ltd., Beijing, China) and stored in a refrigerator at –20°C after qualification verification. The reaction system and conditions used for HYR-PCR detection were as described in sections 2.2.1 and 2.2.2. The NL1/NL4 and ITS1/ITS4 reaction systems were obtained from Zhang et al. (2021).

## 3 Results

### 3.1 Optimization annealing temperature of nested HYR-PCR

**Figure 1:**
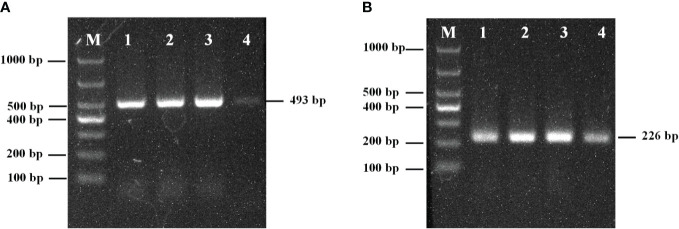
**(A)** The optimum annealing temperature of primers P1/P2; **(B)** The optimum annealing temperature of primers PN1/PN2; M: Marker; 1: 45°C; 2: 50°C; 3: 55°C; 4: 60°C.

The amplification results for the external primer pair P1/P2 are shown in **Figure 1A**. The length of the amplified product fragment (493 bp) and its sequence were consistent with the design expectations and the target sequence. A single bright band was obtained at annealing temperatures of 45, 50, and 55°C, and the brightness increased with increasing temperature; however, the band was faint and indistinct at 60°C annealing temperature. Therefore, 55°C was selected as the optimal annealing temperature.

The amplification results of the internal primers PN1/PN2 are shown in **Figure 1B**. The length of the amplified fragment (226 bp) and its sequence were consistent with design expectations and the target sequence. Bands were observed at 45, 50, 55, and 60°C, but the band brightness was highest at 55°C. Therefore, the optimal annealing temperature for primers PN1/PN2 was 55°C.

### 3.2 Specificity identification

**Figure 2:**
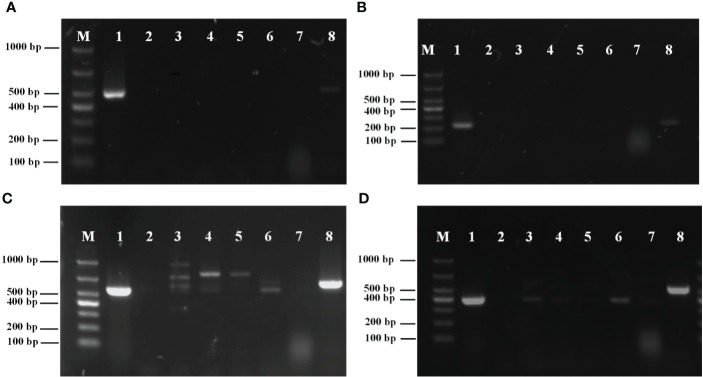
**(A)** Specificity analysis of primers P1/P2; **(B)** Specificity analysis of primers PN1/PN2; **(C)** Specificity analysis of primers NL1/NL4; **(D)** Specificity analysis of primers ITS1/ITS4. M, marker; 1: *Metschnikowia bicuspidata*; 2: ddH_2_O negative control; 3: *Enterocytozoon hepatopenaei*; 4: *Hepatospora eriocheir*; 5: white spot syndrome virus; 6: *Staphylococcus aureus*; 7: *Microsporidia* sp.; 8: *Vishniacozyma victoriae*.

The amplification results for the external primers P1/P2 are shown in **Figure 2A**. The results showed that a single bright band with a fragment length of 493 bp was amplified from *M. bicuspidata*, whereas other pathogens had no specific amplification.

The amplification results for the internal primers PN1/PN2 are shown in **Figure 2B**. A single bright band with a fragment length of 226 bp was amplified from *M. bicuspidata*, whereas other pathogens had no specific amplification.

The amplification results of the conventional PCR primers NL1/NL4 from LSU rRNA are shown in **Figure 2C**. The results showed that primers NL1/NL4 not only amplified from *M. bicuspidata*, but also from *E. hepatopenaei*, *H. eriocheir*, *S. aureus*, white spot syndrome virus, and *V. victoriae*. Moreover, the band position for *S. aureus* was the same as that for *M. bicuspidata*.

The amplification results of the conventional PCR primers ITS1/ITS4 from ITS are shown in **Figure 2D**. Primers ITS1/ITS4 not only amplified the template of *M. bicuspidata*, but also amplified *E. hepatopenaei*, *S. aureus*, and *V. victoriae*, and the band positions for *E. hepatopenaei* and *S. aureus* were the same as that for *M. bicuspidata*.

The results demonstrated that nested HYR-PCR was not disturbed by other pathogens used in this study, and could specifically amplify *M. bicuspidata*.

### 3.3 Nested PCR sensitivity determination

**Figure 3:**
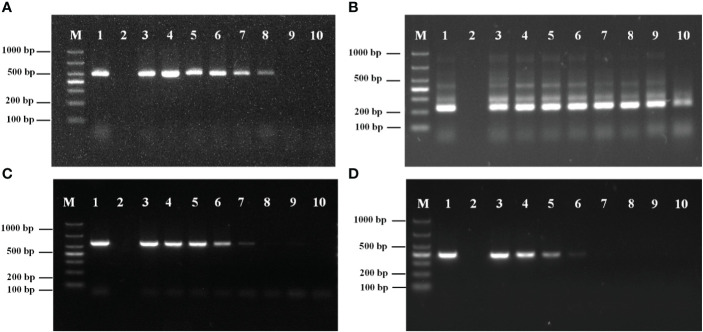
**(A)** Sensitivity analysis of primers P1/P2. **(B)** Sensitivity analysis of primers PN1/PN2. M: Marker; 1: DNA of *Metschnikowia bicuspidata*; 2: ddH_2_O; 3: 6.10×10^8^ copies/μL; 4: 6.10×10^7^ copies/μL; 5: 6.10×10^6^ copies/μL; 6: 6.10×10^5^ copies/μL; 7: 6.10×10^4^ copies/μL; 8: 6.10×10^3^ copies/μL; 9: 6.10×10^2^ copies/μL; 10: 6.10×10^1^ copies/μL. **(C)** Sensitivity analysis of primers NL1/NL4. M: Marker; 1: DNA of *Metschnikowia bicuspidata*; 2: ddH_2_O; 3: 6.03×10^8^ copies/μL; 4: 6.03×10^7^ copies/μL; 5: 6.03×10^6^ copies/μL; 6: 6.03×10^5^ copies/μL; 7: 6.03×10^4^ copies/μL; 8: 6.03×10^3^ copies/μL; 9: 6.03×10^2^ copies/μL; 10: 6.03×10^1^ copies/μL. **(D)** Sensitivity analysis of primers ITS1/ITS4. M: Marker; 1: DNA of *Metschnikowia bicuspidata*; 2: ddH_2_O; 3: 6.74×10^8^ copies/μL; 4: 6.74×10^7^ copies/μL; 5: 6.74×10^6^ copies/μL; 6: 6.74×10^5^ copies/μL; 7: 6.74×10^4^ copies/μL; 8: 6.74×10^3^ copies/μL; 9: 6.74×10^2^ copies/μL; 10: 6.74×10^1^ copies/μL.

The sensitivity PCR results for the external primers (P1/P2) are shown in **Figure 3A**. The minimum detection limit was 6.10 × 10^3^ copies/μL.

The sensitivity results of the internal primers (PN1/PN2) for the second round of amplification are shown in **Figure 3B**. The minimum detection limit was 6.10 × 10^1^ copies/μL.

The amplification results for the conventional primer pairs (NL1/NL4, ITS1/ITS4) are shown in **Figures 3C, D**. The minimum detection limits for NL1/NL4 and ITS1/ITS4 were 6.03 × 10^4^ copies/μL and 6.74 × 10^5^ copies/μL, respectively. The results demonstrated that the sensitivity of the nested HYR-PCR was higher than that of conventional rDNA-PCR.

### 3.4 Clinical samples testing

In 90 clinical samples, specific fragments were amplified from 34 crab samples in the first round of nested HYR-PCR amplification with primers P1/P2, and the infection detection rate was 37.8%.

Specific fragments were amplified from 64 crab samples in the second round of nested PCR amplification with primers PN1/PN2, and the infection detection rate was 71.1%.

Target fragments were amplified from only 15 crab samples using the conventional PCR with primers NL1/NL4 and the infection detection rate was 16.7%. Target fragments were amplified from 22 crab samples using primers ITS1/ITS4, and the infection detection rate was 24.4%. Multiple non-specific amplification products were also observed with primers NL1/NL4 and ITS1/ITS4.

## 4 Discussion

*M. bicuspidata* has caused serious damage to China’s *E. sinensis* culture industry. In recent years, this disease has spread across the country and has been infecting an increasing number of species. Since the report of *M. bicuspidata* infection in *E. sinensis* in 2021 (Bao et al., 2021), infections have also been found in *P. sinensis* and *E. carinicauda* (Cao et al., 2022; Zhao et al., 2022) with the same symptoms as those seen in infected *E. sinensis*, which lead to non-coagulation and milky hemolymph. This indicates that the presence of *M. bicuspidata* in the environment poses a threat to an increasing number of crustaceans. Moore and Strom (2003) found that *M. bicuspidata* could also be transmitted to chinook salmon through *Artemia franciscana*, indicating that the pathogen has a variety of hosts and can spread through the food chain. Therefore, accurate and early detection of pathogen carriers in the environment is important to prevent transmission and outbreaks of this pathogen. The development of rapid, specific, and sensitive molecular biological diagnosis methods is of great significance for eliminating infected *E. sinensis* and blocking the transmission route. Existing pathogen detection methods, such as observations of pathological symptoms, physiological and biochemical characteristics of pathogens, microscopic observation, and conventional PCR, have poor specificity and low sensitivity and are not effective for the accurate detection of *M. bicuspidata* infection (Bao et al., 2021; Zhang et al., 2021).

In this study, we developed a new and specific nested HYR-PCR method for the detection of *M. bicuspidata*, which is based on the hyphally regulated cell wall protein gene. The spore wall of yeast provides environmental protection and participates in host–pathogen interactions through the species-specific spore wall protein, HYR (Bailey et al., 1996; Ebanks et al., 2006; Klis et al., 2014). During the life cycle of *M. bicuspidata*, ascospores attack the gut membrane when *Daphnia* is filter-feeding. Subsequently, surviving spores escape the host hemocyte response and develop into hyphae and sporocysts, and release conidia to complete the infection process (Merrill and Cáceres, 2018). Therefore, the hyphal development stage plays an important role in the infection and life cycle of *M. bicuspidata* (Merrill et al., 2021a; Merrill et al., 2021b). HYR is the main hyphal spore wall protein of yeast fungi, with a length of approximately 349 amino acids. Owing to its important role in the yeast life cycle, its key functional domains (motifs) are highly conserved within *M. bicuspidata* under selective pressure, and the amino acid sequence identity with other yeast species is less than 40% (**Table 5**). Therefore, HYR is a candidate gene locus for the molecular detection of pathogenic yeasts.

**Table 5:** HYR amino acid sequence used for multiple sequence alignment analysis.

Yeast species	% Identity	Accession No.
*Metschnikowia bicuspidata*	–	XP_018712964.1
*Metschnikowia aff. pulcherrima*	37.31	QBM88048.1
*Metschnikowia* sp.	36.61	GEQ71150.1
*Metschnikowia persimmonesis*	37.24	KAF7998685.1
*Candida intermedia*	35.38	SGZ46884.1
*Candida haemuloni* var. *vulneris*	31.69	KAF3986160.1
*Candida pseudohaemulonii*	34.63	XP_024712318.1
*Metschnikowia* sp.	32.94	GEQ69400.1
*Debaryomyces hansenii*	32.74	XP_002770057.1
*Candida haemuloni*	32.83	XP_025342839.1
*Candida auris*	32.50	QRG37544.1

Nested HYR-PCR was superior to rDNA-PCR in terms of its specificity and sensitivity. In this study, two primer pairs targeting the HYR gene showed very strong specificity and clear bands during sample analysis. In contrast, the primers designed for the LSU rRNA and ITS regions also amplified template DNA from other pathogens, and for *S. aureus*, the target band appeared at the same position as that for *M. bicuspidata*. This can lead to false detections if other microorganisms are present in environmental samples or individuals, and sequencing of the target bands is necessary to confirm the species identity. The two pairs of nested HYR-PCR primers showed no specific amplification with the other six pathogens tested. Therefore, any specific amplification in this study confirmed the presence of *M. bicuspidata*, and this was verified in the detection of clinical samples. The sensitivity experiment showed that the nested HYR-PCR had much greater sensitivity (6.10 × 10^1^ copies/μL) than that found with the LSU rRNA (6.03 × 10^4^ copies/μL) and ITS (6.74 × 10^5^ copies/μL) primers.

The clinical samples experiment confirmed that the positivity rate of the nested HYR-PCR was much higher than that of the conventional PCR. Nested HYR-PCR detected 64 positive results in 90 clinical samples (71.1%), which sequence alignment analysis confirmed to be fragments of the *M. bicuspidata* HYR gene. In contrast, the positivity rates obtained using the LSU rRNA and ITS primers were only 16.7% and 24.4%, respectively. Moreover, even the first round of the nested HYR-PCR (positivity rate 37.8%) showed higher sensitivity than that of conventional PCR, and there was no nonspecific amplification, which further demonstrated that the screened primers had high specificity and good sensitivity. Additionally, the nonspecific amplification of diagnostic methods happens to small subunit ribosomal RNA genes (SSU rRNA) (Lucchi et al., 2012; Hitakarun et al., 2014; Jaroenlak et al., 2016). For example, in single-step PCR detection of *E. hepatopenaei*, SSU rRNA primers can cross-amplify with other microsporidia present in aquatic animals (Jaroenlak et al., 2016). Similarly, the single-step PCR detection method for *Leishmania siamensis* based on the SSU rRNA gene showed cross-amplification with *Trypanosoma brucei* and *T. Evans* (Hitakarun et al., 2014). Therefore, for environmental samples, it is necessary to use the nested HYR-PCR method or sequencing to confirm the identity of *M. bicuspidata* infecting suspected carriers with rDNA-PCR positivity. The gene sequence we referenced was produced from the *M. bicuspidata* strain in the U.S. and the detection sample in this study was from the strain in the Chinese mitten crab. Although the strains were obtained from different sources, they still showed good specificity, indicating that the detection method is largely practical. However, how the HYR gene polymorphism of *M. bicuspidata* is still unclear, and the verification of more strains is still needed to avoid false negatives due to possible sequence changes.

In conclusion, we developed a new nested HYR-PCR method for the detection of *M. bicuspidata* infections. Compared with previous methods, the nested HYR-PCR method has higher specificity and sensitivity, and is suitable for the detection of *M. bicuspidata* in clinical crab samples. The nested HYR-PCR method will be a useful tool for studying the transmission route of *M. bicuspidata* and will facilitate the design of more effective management and control measures for *M. bicuspidata* disease.

## Data availability statement

The original contributions presented in the study are included in the article/supplementary material. Further inquiries can be directed to the corresponding author.

## Ethics statement

The animal study was reviewed and approved by Animal Experiments Ethics Committee of Shenyang Agricultural University.

## Author contributions

JB, and HJ were involved in designing of the experiment and wrote the manuscript. YC, JB, CF, and HJ performed the majority of the experiment, data analysis and interpretation. YX, QH, and XL assisted in sample collection and primer screening. All authors contributed to the article and approved the submitted version.

## Funding

This research was funded by Liaoning province “The Open Competition Mechanism to Select the Best Candidates” Project (2021JH1/10400040); China Agriculture Research System of MOF and MARA (CARS-48); Shenyang Science and Technology mission project (21-116-3-38); Liaoning province Department of Education fund item (LSNQN202002).

## Conflict of interest

The authors declare that the research was conducted in the absence of any commercial or financial relationships that could be construed as a potential conflict of interest.

## Publisher’s note

All claims expressed in this article are solely those of the authors and do not necessarily represent those of their affiliated organizations, or those of the publisher, the editors and the reviewers. Any product that may be evaluated in this article, or claim that may be made by its manufacturer, is not guaranteed or endorsed by the publisher.
